# Insights into the genetic architecture of *Phytophthora capsici* root rot resistance in chile pepper (*Capsicum* spp.) from multi-locus genome-wide association study

**DOI:** 10.1186/s12870-024-05097-2

**Published:** 2024-05-17

**Authors:** Navdeep Kaur, Dennis N. Lozada, Madhav Bhatta, Derek W. Barchenger, Ehtisham S. Khokhar, Seyed Shahabeddin Nourbakhsh, Soum Sanogo

**Affiliations:** 1 Department of Plant and Environmental Sciences, New Mexico State University, Las Cruces, NM 88003 USA; 2 Current address: Department of Horticultural Sciences, Texas A&M University, College Station, TX 77843 USA; 3 Chile Pepper Institute, New Mexico State University, Las Cruces, NM 88003 USA; 4 Bayer Crop Science, Chesterfield, MO 63017 USA; 5 World Vegetable Center, Shanhua, Tainan, 74151 Taiwan; 6 Department of Extension Plant Sciences, New Mexico State University, Las Cruces, NM 88003 USA; 7 Department of Entomology, Plant Pathology and Weed Science, New Mexico State University, Las Cruces, NM 88003 USA

**Keywords:** Association genetics, Candidate genes, Disease Resistance, Genotyping-by-sequencing, Multi-locus random mixed linear models, Single nucleotide polymorphisms

## Abstract

**Background:**

Phytophthora root rot, a major constraint in chile pepper production worldwide, is caused by the soil-borne oomycete, *Phytophthora capsici*. This study aimed to detect significant regions in the *Capsicum* genome linked to Phytophthora root rot resistance using a panel consisting of 157 *Capsicum* spp. genotypes. Multi-locus genome wide association study (GWAS) was conducted using single nucleotide polymorphism (SNP) markers derived from genotyping-by-sequencing (GBS). Individual plants were separately inoculated with *P. capsici* isolates, ‘PWB-185’, ‘PWB-186’, and ‘6347’, at the 4–8 leaf stage and were scored for disease symptoms up to 14-days post-inoculation. Disease scores were used to calculate disease parameters including disease severity index percentage, percent of resistant plants, area under disease progress curve, and estimated marginal means for each genotype.

**Results:**

Most of the genotypes displayed root rot symptoms, whereas five accessions were completely resistant to all the isolates and displayed no symptoms of infection. A total of 55,117 SNP markers derived from GBS were used to perform multi-locus GWAS which identified 330 significant SNP markers associated with disease resistance. Of these, 56 SNP markers distributed across all the 12 chromosomes were common across the isolates, indicating association with more durable resistance. Candidate genes including nucleotide-binding site leucine-rich repeat (NBS-LRR), systemic acquired resistance (SAR8.2), and receptor-like kinase (RLKs), were identified within 0.5 Mb of the associated markers.

**Conclusions:**

Results will be used to improve resistance to Phytophthora root rot in chile pepper by the development of Kompetitive allele-specific markers (KASP®) for marker validation, genomewide selection, and marker-assisted breeding.

**Supplementary Information:**

The online version contains supplementary material available at 10.1186/s12870-024-05097-2.

## Background

*Phytophthora capsici*, a soil-borne oomycete first isolated in 1918 from chile pepper plants at New Mexico Agricultural Experiment Station field plots, and formally described in 1922 [1], remains a major problem in chile production worldwide [2, 3]. The pathogen infects multiple parts of the chile pepper plant, including stems, roots, fruits, and leaves at all stages of growth, and can cause significant production losses up to 100% [2, 4]. The most destructive disease symptom of *P. capsici* is root rot. Damping off due to root rot can cause death within two to five days in seedlings after infection; root rot can result in stunted growth, wilting, and ultimately plant death in older plants within approximately two weeks [2, 5, 6].

Various management practices including crop rotation, water management, fumigation, soil solarization, and fungicide application have been used but none of these strategies have been completely effective in controlling the disease [2, 7]. The most efficient, eco-friendly, and sustainable approach to managing this pathogen is through the utilization of resistant cultivars [8, 9]. The complexity of the *Phytophthora-Capsicum* pathosystem is a major challenge in disease resistance breeding. Different resistant genes are known to be required for resistance to different physiological races and syndromes [8, 10–12]. Moreover, several inheritance models including single dominant [6] and two-gene or multiple genes with dominant and recessive epistasis [2, 8] have been reported for *P. capsici* resistance, which presents an additional challenge. A dominant inhibitor for *P. capsici* resistance (*Ipcr*) gene which inhibits polygenic host resistance has also been identified in chile peppers [13]. Many resistant accessions have been found with different levels of resistance to various isolates. Among the different sources identified, the Mexican landrace, ‘Criollo de Morelos-334’ (‘CM-334’), has been known to have the greatest degree of resistance to all the physiological races of *P. capsici* and has been widely utilized in various breeding programs [9, 14]. To this date, however, a resistant commercial cultivar with broad-spectrum resistance comparable to the original resistant sources has not been developed [9].

Genetic studies have mainly focused on traditional biparental linkage analyses for various complex traits in chile pepper such as capsaicinoid content [15–17], yield-related traits [18, 19], fruit-related traits [20–22], plant architecture [21, 23], cucumber mosaic virus (CMV; *Cucumovirus*) resistance [24, 25], and *P. capsici* resistance [8, 9, 26–28]. This mapping approach depends on the genetic variation found in the parents and can only identify broad genomic regions, making it challenging to detect the specific candidate genes responsible [16]. Genomewide association study (GWAS) is an efficient tool that can be utilized to identify genetic loci linked with various complex traits using natural populations [29] and can be used to complement biparental mapping. Previously, a total of 117 and 30 significant SNP markers were identified across the entire *Capsicum* genome using single-locus GWAS for *P. capsici* resistance using 352 [9] and 342 accessions [30].

The current study utilized multi-locus GWAS models which allowed simultaneous calculation and testing of marker effects, improving its power over single-locus GWAS models [31, 32]. Various crop species have already employed multi-locus GWAS models including wheat (*Triticum aestivum*) [33–35], corn (*Zea mays*) [36–38], soybean (*Glycine max*) [39–41], rice (*Oryza sativa*) [42, 43], and barley (*Hordeum vulgare*) [44, 45] to understand the genetics of different complex traits. Although multi-locus GWAS models have been applied recently in chile pepper to identify genomic regions associated with yield, yield components, and other agronomic traits [32], there is currently no existing report on their implementation for the genetic dissection of resistance to *P. capsici* root rot in *Capsicum*.

The primary goal of this study was to understand the genetic basis of Phytophthora root rot resistance using a diverse population of *Capsicum* accessions. The specific objectives were to (1) screen a diverse panel of chile pepper for resistance to three different isolates (‘PWB-185’, ‘PWB-186’, and isolate ‘6347’) of *P. capsici* with varying degrees of virulence, (2) identify significant genomic regions linked to *Phytophthora* root rot resistance using multi-locus GWAS models, and (3) determine candidate genes associated with these significant loci. Results from this research will be relevant for molecular breeding for improving Phytophthora root rot resistance in chile pepper.

## Results

### Disease resistance screening

The diversity panel was screened for resistance to *P. capsici* isolates with varying degrees of virulence, viz. ‘PWB-185’ (moderate virulence), ‘PWB-186’ (low virulence), and ‘6347’ (high virulence). The resistant check (‘CM-334’) showed no symptoms of infection when screened against each of the isolates. The susceptible checks (‘Camelot’ and ‘NMCA 10399’) inoculated with ‘PWB-185’ started showing symptoms at 5-days post-inoculation (DPI) and were completely wilted and dead at 14-DPI. For isolate ‘6347’, the susceptible checks ‘Ninja’ and ‘NMCA 10399’, started displaying symptoms at 5-DPI and were wilted and dead by 10-DPI. The susceptible checks ‘Ninja’ and ‘NMCA 10399’ inoculated with ‘PWB-186’ started showing symptoms at 7–10-DPI, but the complete death of plants was not detected at 14-DPI, when the bioassay was completed.

**Fig. 1:**
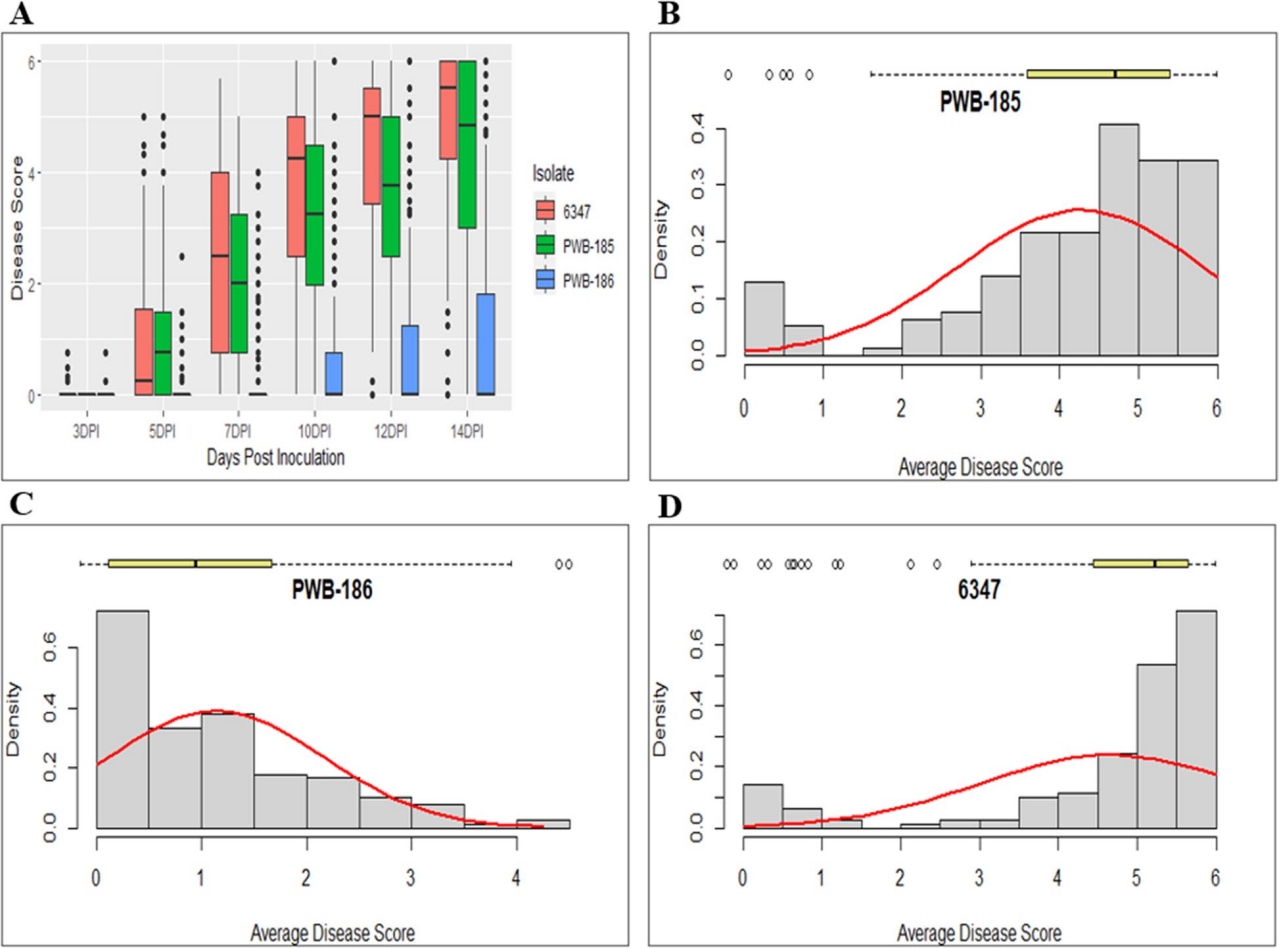
**A** Disease progression in *Capsicum* accessions inoculated with three different isolates of *Phytophthora capsici*. **B** Distribution of the average disease scores at 14-DPI for isolate ‘PWB-185’, (**C**) ‘PWB-186’, and (**D**) ‘6347’. The test for normality overlaid with a red line

The plants were scored every day on a 0–6 disease rating scale starting at 3-DPI [8, 46] up to 14-DPI (Fig. 1A). For both ‘PWB-185’ and ‘6347’ isolates, 90.4% and 84.1% accessions started showing symptoms at 5-DPI respectively; however, for ‘PWB-186’, only 24.2% of the accessions started showing symptoms at 5-DPI. Out of 157 accessions in the diversity panel, eight were completely resistant (disease score 0 at 14-DPI) to isolate ‘PWB-185’ and 35 accessions were not significantly different from the resistant check (‘CM-334’) (*P* ≥ 0.05). For ‘PWB-186’, 33 accessions in the diversity panel were completely resistant (disease score 0 at 14-DPI), and 130 accessions were not significantly different from the resistant check (*P* ≥ 0.05). Seven accessions were completely resistant to isolate ‘6347’ and 27 were not significantly different from the resistant check for this isolate (*P* ≥ 0.05). Overall, five *C. annuum* lines in the diversity panel, namely, ‘Chilhuacle Orange’, ‘Tipo Ancho’, ‘NMCA 10237’, ‘13C905-6’, and ‘Tipo Pasilla’ were completely resistant to the three isolates.

Significant differences were revealed using analysis of variance (ANOVA) between entries, replications, DPI, and blocks nested in replications for each isolate (*P* < 0.0001) (Table 1). Entry and DPI interactions were also tested in ANOVA and significant interactions were detected for isolates ‘PWB-185’ and ‘6347’ (*P* < 0.0001), but not for isolate ‘PWB-186’. Average disease score distribution was left-skewed (towards susceptibility) for ‘PWB-185’ and ‘6347’ and it was right-skewed (towards resistance) for ‘PWB-186’ (Shapiro–Wilk test, *P* < 0.0001; Fig. 1 B, C, D).

**Table 1:** Analysis of variance (ANOVA) for the three *Phytophthora capsici* isolates used in the study. Significant differences were detected in days post-inoculation (DPI), replications, blocks nested in replications, and entries (*P* < 0.0001). A significant interaction between entries and days post-inoculation was detected for isolates ‘PWB-185’ and ‘6347’

Isolate	Source		Df	Sum Sq	Mean Sq	F value	Pr(> F)
**PWB-185**		Entry	173	4421	25.6	26.53	< 2e-16^***^
		Replication	2	171	85.4	88.63	< 2e-16^***^
		DPI	5	7379	1475.7	1532.06	< 2e-16^***^
		Block (Replication)	33	319	9.7	10.03	< 2e-16^***^
		Entry:DPI	865	1778	2.1	2.13	< 2e-16^***^
		Residuals	2639	2542	1.0		
**PWB-186**		Entry	165	718	4.35	5.573	< 2e-16^***^
		Replication	2	186.2	93.11	119.26	< 2e-16^***^
		DPI	5	545.6	109.13	139.77	< 2e-16^***^
		Block (Replication)	30	217.9	7.26	9.30	< 2e-16^***^
		Entry:DPI	825	518.6	0.63	0.81	1^NS^
		Residuals	2476	1933.2	0.78		
**6347**		Entry	163	5289	32.4	39.01	< 2e-16^***^
		Replication	2	292	146.2	175.70	< 2e-16^***^
		DPI	5	9291	1858.2	2233.79	< 2e-16^***^
		Block (Replication)	30	479	16.0	19.21	< 2e-16^***^
		Entry:DPI	815	2160	2.7	3.19	< 2e-16^***^
		Residuals	2476	2060	0.8		

^***^Significant at *P* < 0.0001; **Significant at *P* < 0.001; *Significant at *P* < 0.01; *NS* Not significant

The highest average AUDPC values for ‘PWB-185’ were 46.2 (‘NMCA 11322’) and 45.9 (‘Mesilla’). Susceptible checks ‘Camelot’ and ‘NMCA 10399’ had average AUDPC values of 40.2 and 37.8, respectively. A total of 46 accessions had disease severity index percentage (DSI%) greater than 90%, and 11 accessions had resistant plants percentages greater than 90% against ‘PWB-185’. For the less virulent ‘PWB-186’ isolate, the highest average AUDPC values were 20.9 (‘20C239’) and 20.7 (‘Mallorca Paprika’), whereas the susceptible checks ‘Ninja’ and ‘NMCA 10399’ had average AUDPC values of 3.4 and 3.2, respectively. There were no accessions with DSI% greater than 90%, when screened using ‘PWB-186’, whereas 49 accessions had resistant plants percentages greater than 90%. The highest average AUDPC values for isolate ‘6347’ were 54.1 (‘NMCA 10406’) and 54.0 (‘Large Red Thick Cayenne’). The susceptible checks ‘Ninja’ and ‘NMCA 10399’ had average AUDPC values of 37.14 and 45.9, respectively. The number of accessions with DSI% and percentage of resistant plants greater than 90%, against ‘6347’ were 77 and 11, respectively. The broad-sense heritability (*H*^*2*^) for Phytophthora root rot resistance varied among the isolates and was related to the level of virulence. *H*^*2*^ values for the traits were higher for the more virulent isolates, with an average of 0.78 and 0.90 for isolates ‘PWB-185’ and ‘6347’, respectively. For the ‘PWB-186’ isolate, *H*^*2*^ across the traits had an average value of 0.25 (Table 2).

**Table 2:** Genotypic and phenotypic variance and broad sense heritability values for the disease parameters for the three *Phytophthora capsici* isolates

Isolate	Parameter	Genotypic variance (*Vg*)	Phenotypic variance (*Vp*)	Heritability (*H*^*2*^)
**PWB-185**	Disease Score	1.94	2.46	0.78
	AUDPC	91.90	122.14	0.75
	DSI%	537.92	684.40	0.78
	Percent resistant plants	594.51	742.30	0.80
**PWB-186**	Disease Score	0.23	1.0148	0.22
	AUDPC	8.20	26.19	0.31
	DSI%	63.16	281.88	0.22
	Percent resistant plants	118.68	517.77	0.23
**6347**	Disease Score	2.65	2.88	0.92
	AUDPC	146.61	179.22	0.82
	DSI%	737.35	800.47	0.92
	Percent resistant plants	818.51	864.61	0.95

### GBS-derived SNP markers

**Fig. 2:**
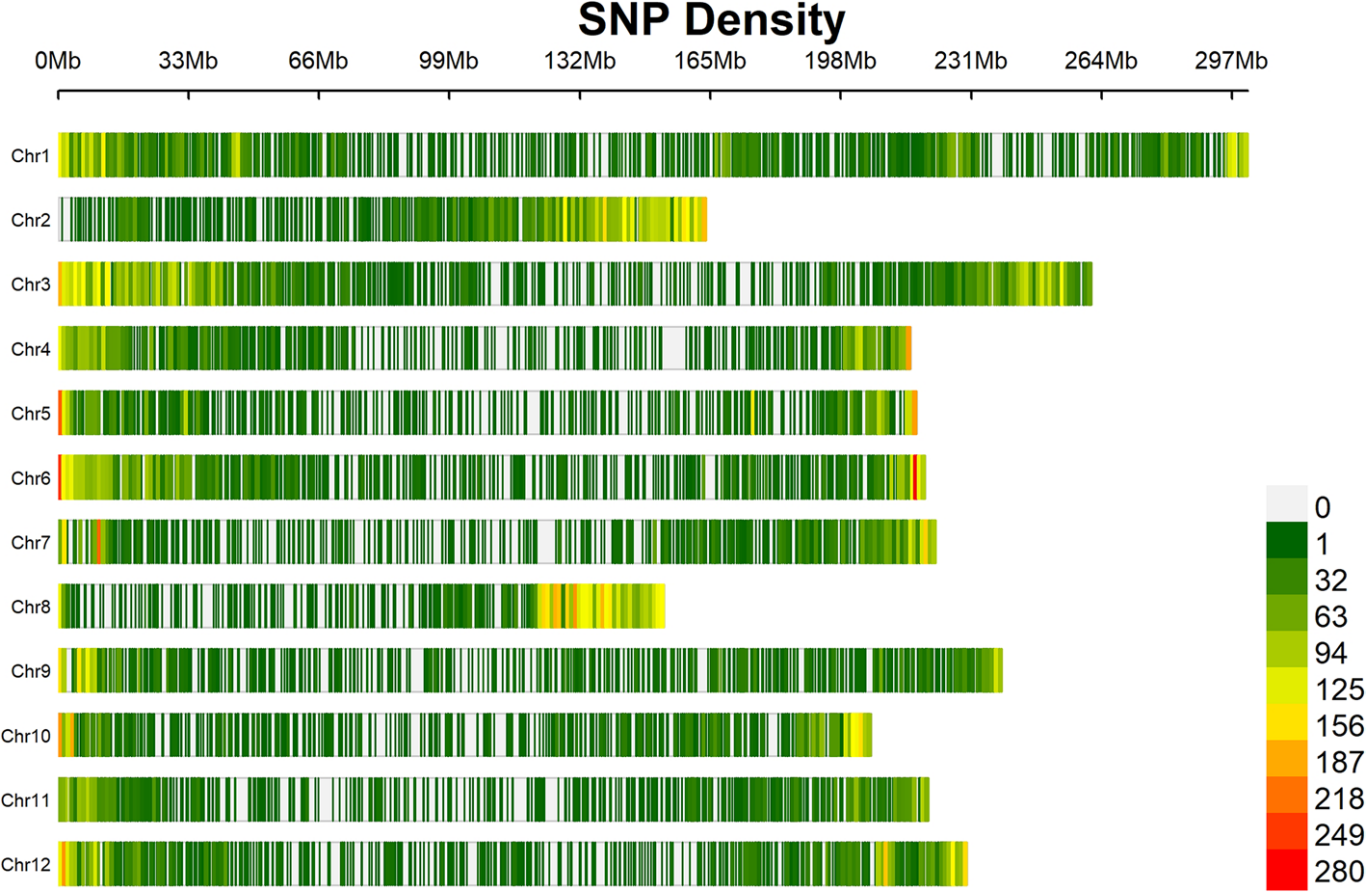
Chromosome wise single nucleotide polymorphism (SNP) density plot with the number of SNP markers within a 1 Mb window. The horizontal axis represents the length of the chromosomes, and the bars represent chromosomes 1 to 12

The original genotype file obtained after the sequence analysis had a total of 404,188 SNP markers. Excluding minor SNP states and SNPs with < 0.05 minor allele frequency resulted in 61,714 SNP markers. After filtering out the unmapped markers, 55,117 SNP loci with known chromosomal locations were identified and were used for multi-locus GWAS. The most predominant nucleotide was adenine (‘A’) (23.74%), followed by guanine (‘G’) (23.4%), thymine (‘T’) (23.2%), and cytosine (‘C’) (22.8%). The most common substitution types were ‘C/T’ transition (8,700; 14.09%) and ‘G/A’ transition (8,680; 14.06%). The most common transversion was ‘A/T’ (4,296; 6.96%) (Table 3). Chromosome 3 had the most markers with 7,388, followed by chromosomes 1 with 6,113 and 2 with 5,437. Chromosome 11 had the fewest markers with 3,217, followed by chromosomes 5 with 3,517 and 10 with 3,624. Chromosome 2 had the highest SNP density (33.2 SNPs/Mb), followed by chromosomes 8 (30.6 SNPs/Mb) and 3 (28.3 SNPs/Mb); whereas chromosome 11 had the least marker density of 14.6 SNPs/Mb, followed by chromosomes 9 (15.54 SNPs/Mb) and 5 (16.2 SNPs/Mb) (Fig. 2). The overall marker density for the whole genome was (20.8 SNPs/Mb).

**Table 3:** Allele summary for the SNP markers obtained for the chile pepper population

Allele	Number of SNP sites	Frequency
A	1,867,009	0.24
G	1,838,110	0.23
T	1,8255,40	0.23
C	1,793,056	0.23
C/T (Transition)	8,700	0.14
G/A (Transition)	8,680	0.14
A/G (Transition)	7,795	0.13
T/C (Transition)	7,268	0.12
A/T (Transversion)	4,296	0.07
T/A (Transversion)	4,190	0.07
G/T (Transversion)	4,160	0.07
C/A (Transversion)	3,853	0.06
T/G (Transversion)	3,707	0.06
A/C (Transversion)	3,518	0.06
C/G (Transversion)	2,821	0.05
G/C (Transversion)	2,726	0.04

### Population structure and linkage disequilibrium

**Fig. 3:**
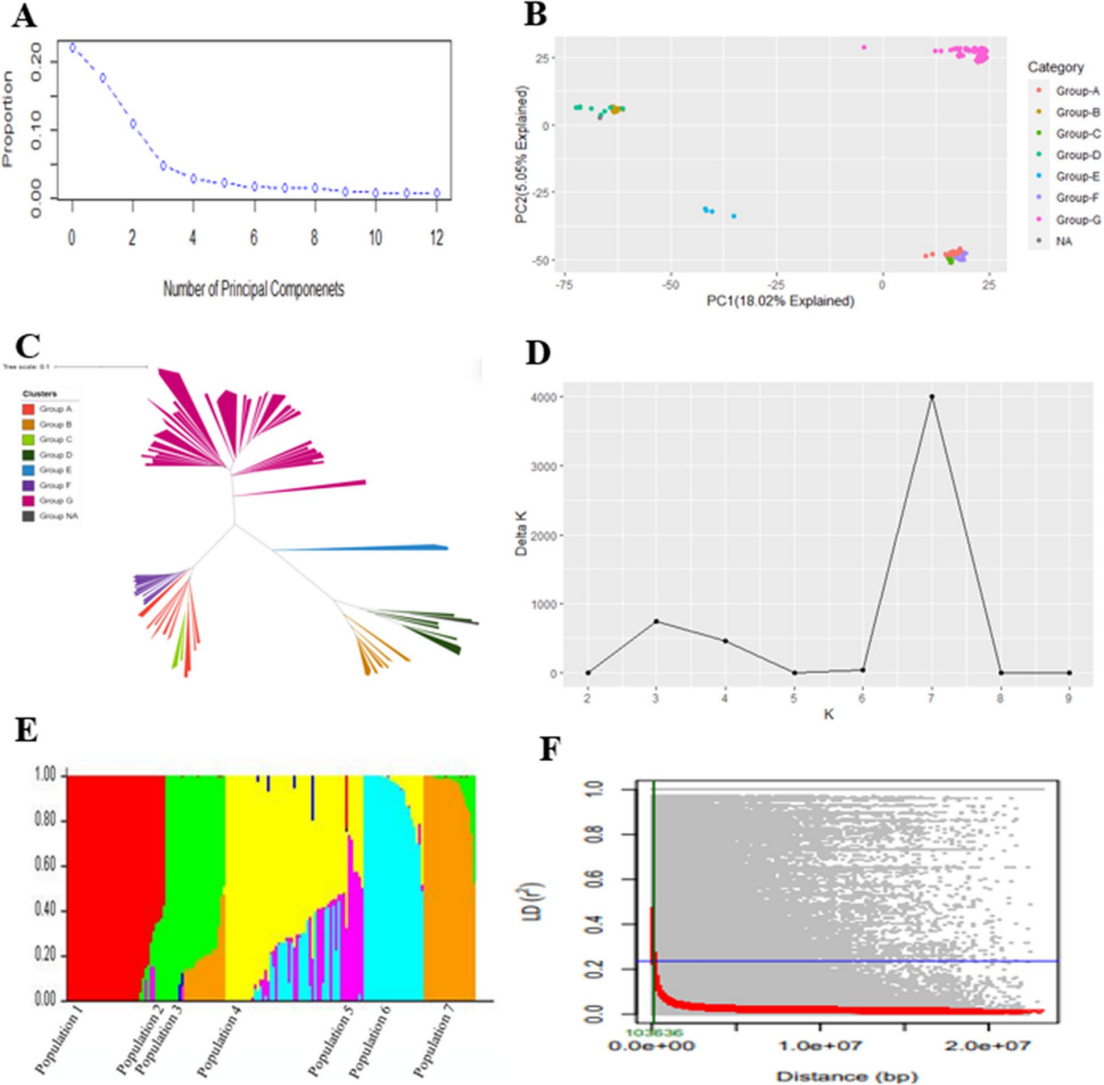
**A** Scree plot illustrating the gradual decline in the proportion of variance explained with the increase in the number of principal components. **B** PCA biplot constructed using the first two principal components demonstrating the genetic stratification in the diversity panel. **C** Neighbor-joining tree depicts the genetic relatedness of the 157 accessions used in this study. **D** The Evanno method determined the optimal number of clusters for the population, *K* = 7 with the highest value for *ΔK*. **E** Bar plots of the population structure coefficients of the 157 accessions. Seven colors represent seven subpopulations, respectively. An individual is represented by each vertical column and the percentage of the individual in the population is represented by each colored segment in each column. **F** Decay plot for linkage disequilibrium (LD). The half-decay value for LD (*r*.^*2*^ = 0.236) is represented by the blue line and the non-linear regression curve corresponds to the red solid line. The green line corresponds to the distance in the base pair when the LD drops to half of its maximum value (~ 0.10 Mb)

Principal component analysis (PCA) (Fig. 3A) revealed that the proportion of the variance defined by the principal components dropped after the fifth principal component (PC) and almost plateaued. A few of the accessions did not belong to any of the three major clusters, based on the PCA (Fig. 3B). Thus, to account for the structure of the whole population, five PCs were selected for the GWAS analysis. The PC1, PC2, PC3, PC4, and PC5 explained 17.8%, 11.1%, 4.9%, 2.9%, and 2.3% of the genotypic variance, respectively. Seven phylogenetic clusters were revealed using the neighbor-joining tree (Fig. 3C). Similarly, the Evanno criterion determined the optimum number of clusters, *K* = 7 (*ΔK* = 4,005.3)*,* in the population based on STRUCTURE analysis, which suggested that the population could be divided into seven admixed subpopulations (Figs. 3D, E). Cluster 1 (Group A; *N* = 19) consisted of cultivars with a wide range of fruit types including bell, wax, cayenne, de arbol, poblano, serrano, pasilla, mirasol, jalapeño, and chilhuacle. Cluster 2 (Group B; *N* = 20) included breeding lines with New Mexican, cayenne, and paprika fruit types. Cluster 3 (Group C; *N* = 5) consisted of cultivars with jalapeño fruit types and cluster 4 (Group D; *N* = 13) included breeding lines (8) and cultivars (2) with cayenne fruit type and three cultivars with banana or wax pepper, jalapeño, and serrano fruit types. Cluster 5 (Group E; *N* = 4) consisted of interspecific hybrid accessions derived from crossing *C. annuum* with *C. frutescens.* Cluster 6 (Group F; *N* = 19) mostly included cultivars with New Mexican (including paprika types), a landrace, and a breeding line with jalapeño fruit type. Cluster 7 (Group G; *N* = 76) was an admixed of 64 breeding lines and 12 landraces with different fruit types including New Mexican, jalapeño, cayenne, poblano, and the rest included Aleppo, Asian, guajillo, mirasol, paprika, pasilla, pimiento, shipkas, and wax fruit types. The number of clusters, *K* = 3 also serves as an alternative number of optimum clusters as it displayed a high *ΔK* value of 752.47 compared to the other clusters (Fig. 3D).

A total of 1,944,143 intrachromosomal marker pairs were used for LD analysis, and 421,560 pairs (21.7%) were within significant LD (*P* < 0.05). The largest number of marker pairs within significant LD were found on chromosome 3 (61, 440), whereas chromosome 11 had the fewest (22, 047). A total of 174, 621 marker pairs showed complete LD (coefficient of LD, *r*^*2*^ = 1.0). Chromosome 3 had the highest number of marker pairs in complete LD (26,041). The average distance between the pairs of markers in significant LD ranged from 0.63 (chromosome 2) to 1.64 Mb (chromosome 9) (Table S1). The *r*^*2*^ values were observed to decrease over genetic distance (in Mb). Genome wide LD decay rate was approximately 0.10 Mb (Fig. 3F).

### Significant SNPs for *Phytophthora capsici* root rot resistance

**Fig. 4:**
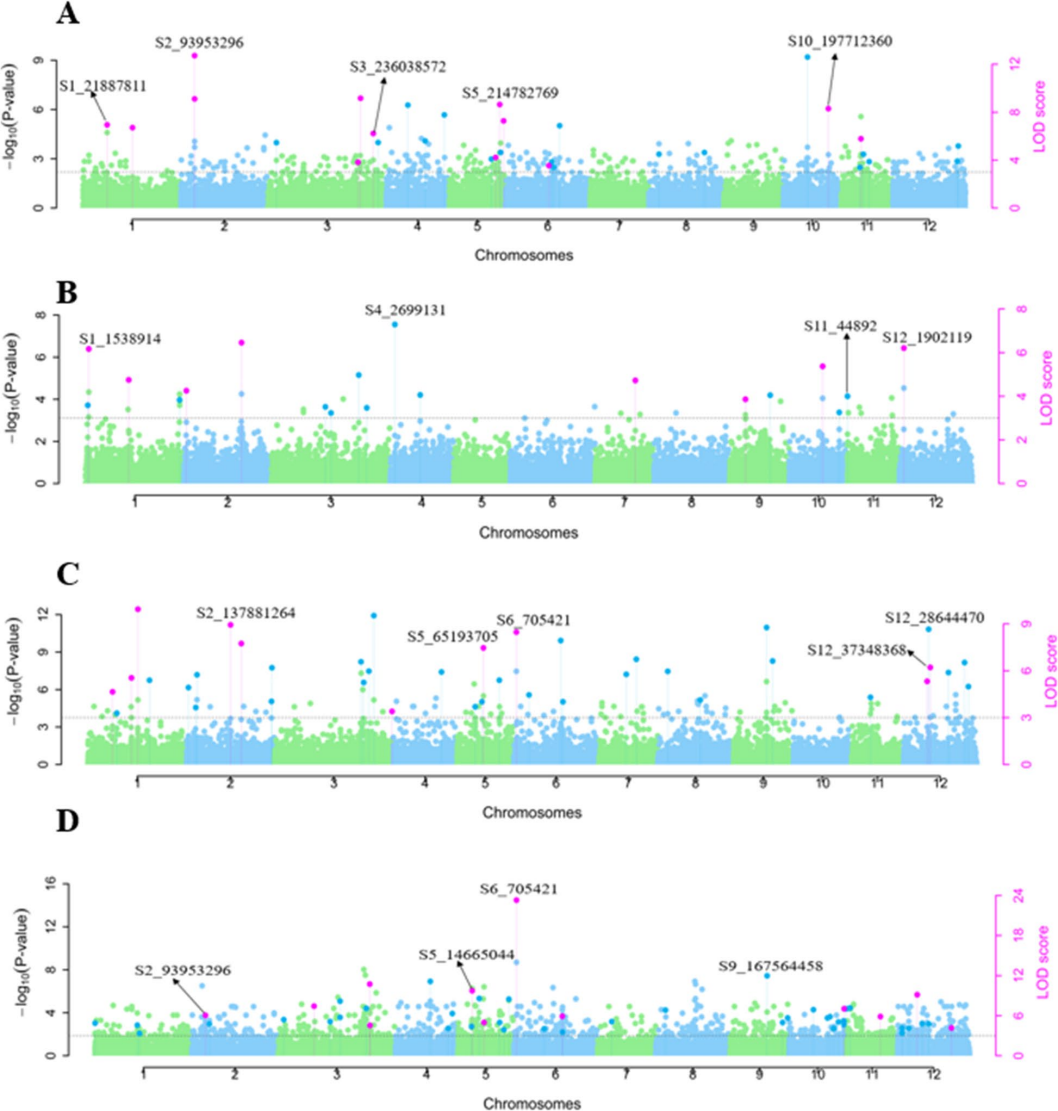
Manhattan plot for average disease scores at 14-DPI for *Phytophthora capsici* (**A**) isolate ‘PWB-185’ BLUEs, (**B**) isolate ‘PWB-186’ BLUEs, (**C**) isolate ‘6347’ BLUEs, and (**D**) BLUPs

Overall, 330 SNP markers were detected to be linked to Phytophthora root rot resistance using different multi-locus GWAS models on various disease parameters (Table S2). For the ‘PWB-185’ isolate, 33, 31, 37, 28, and 34 SNPs were found to be significant (LOD score ≥ 3.0) by all six multi-locus GWAS models for AUDPC, disease score at 14-DPI, DSI%, estimated marginal means, and percent resistant plants, respectively. For the ‘PWB-186’ isolate, 30, 19, 24, 23, and 25 SNPs were significant for AUDPC, disease score at 14-DPI, DSI%, estimated marginal means, and percent resistant plants, respectively. In the case of isolate ‘6347’, 33, 39, 40, 37, and 44 significant SNPs were identified for AUDPC, disease score at 14-DPI, DSI%, estimated marginal means, and percent resistant plants, respectively. Lastly, for BLUPs, 33, 50, 55, 52, and 43 significant SNPs were found using all the multi-locus GWAS methods for AUDPC, disease score at 14-DPI, DSI%, estimated marginal means, and percent resistant plants, respectively. There was a total of 95, 76, 94, and 113 significant SNP markers for the four datasets for average disease score at 14-DPI for the four phenotypic datasets (Fig. 4). In the case of disease parameters, the highest number of total significant markers were identified for DSI% (136), followed by percentage of resistant plants (127). The total significant markers identified for disease score at 14DPI, estimated marginal means, and AUDPC were 123, 124, and 114 respectively. A total of 56 SNP markers were common across all four phenotypic datasets (Table S3).

Only a single SNP marker, *S3_236038572,* present on chromosome 3 was detected in all datasets and accounted for 0.74% to 9.92% of phenotypic variation (Fig. 4A). Out of the 56 significant markers, 11 markers were detected in at least three phenotypic datasets. The maximum number of common significant markers were identified on chromosome 12 (9 SNPs), followed by chromosomes 5 (7) and 6 (7). The maximum phenotypic variation among the common markers was explained by *S6_705421* which was detected in three phenotypic datasets (‘PWB-185’, ‘6347’, and BLUP), ranging from 4.6% to 54.4%. Some of the significant SNPs had positive allele effects for AUDPC, DSI%, disease scores at 14-DPI, and estimated marginal means and had negative allele effects for percentage of resistant plants which indicates their potential association with susceptibility genes. For instance, the allele effect for SNP *S5_65193705* on chromosome 5 was 21.8 for DSI% and -12.20 for percent resistant plants. A total of 38 out of the 56 common significant markers were found to be potentially linked to susceptibility genes. On the other hand, some SNPs had positive allele effects for percentage of resistant plants and negative allele effects for the rest of the disease parameters indicating their association with resistance genes. For example, SNP *S9_167564458* had allele effects ranging from -25.1 for DSI% to 67.6 for percentage of resistant plants. A total of 18 significant SNP markers located on chromosomes 1, 3, 4, 5, 6, 9, 10, 11, and 12 were potentially associated with resistance genes. These potential associations with resistance or susceptibility genes can be confirmed with performing marker and QTL validation studies.

### Candidate genes for *Phytophthora* root rot resistance

The 56 significant markers that were common across all phenotypic datasets were associated with a total of 829 candidate genes (Table S4). These genes are involved in a range of biological functions including cell cycle and division, oxidation–reduction, phosphorylation, defense response, DNA repair, protein serine/threonine kinase activity, metabolic processes, cell wall organization and biosynthesis, among others. A total of 15 genes that code for LRR receptor-like serine/threonine-protein kinase which are well-known for involvement in disease resistance were detected close to six SNPs (*S2_137881264*, *S3_38944749*, *S4_212420204*, *S10_122768586*, *S10_189438330*, and *S12_5016287*). Other genes associated with defense response including late blight resistance protein R1-A, *β*-1,3-glucanases, mitogen-activated protein kinase, elicitor peptide 6, receptor-like proteins, and ethylene responsive transcription factors, were also identified. These genes play a role in different mechanisms of action in defense response and could be good candidates for Phytophthora root rot resistance in *Capsicum*.

The SNP, *S6_705421*, which explained the highest phenotypic variation (*R*^*2*^ = 54.4%), had 47 candidate genes in its proximity, which included four putative late blight resistance protein coding genes, one gene coding for leucine rich repeat domain and one gene that codes for NB-ARC domain-containing protein. A systemic acquired resistance (SAR 8.2) protein coding gene was detected 471 kb downstream to the significant SNP, *S5_14665044*, located on chromosome 5. Another SNP *S2_137881264* on chromosome 2 with an LOD value up to 13.74 had a total of 52 genes within 0.5 Mb region upstream and downstream. It included one late blight resistance gene and nine putative LRR receptor-like serine/threonine-protein kinase coding genes.

## Discussion

Phytophthora root rot is a highly damaging disease of chile pepper present throughout the globe and is very difficult to manage [2]. With modern molecular plant breeding tools, disease resistance QTL and genes can be detected, which ultimately could contribute to the development of resistant chile pepper varieties. GWAS is a tool used in plant breeding which provides higher mapping resolution than traditional biparental mapping approaches [47]. This study employed multi-locus GWAS approaches to better understand the genetics of resistance to *P. capsici* root rot in chile pepper. The virulence of *P. capsici* isolates used for screening exhibited variation, where some genotypes displayed resistance to specific races. A complex genetic relatedness was observed within the *Capsicum* population used for screening *P. capsici* root rot. Further, the complex genetic basis of disease resistance is reflected by the diversity of candidate genes identified in this study.

### Variation in virulence for the *P. capsici* isolates and race-specific resistance

The genotypes that are virulent to the same host are referred to as a physiological race of a pathogen [48]. This study involved three isolates of *P. capsici*, each of which belonged to different physiological races with different levels of virulence. Around 82.8% of the accessions screened with isolate ‘PWB-186’ showed no significant difference in the average disease ratings compared to the resistant control (‘CM-334’). For isolates ‘PWB-185’ and ‘6347’, 22.3% and 17.2% of the accessions, respectively, did not exhibit significant differences from the resistant control. The susceptible checks for ‘PWB-186’ were not dead at 14-DPI, whereas for ‘PWB-185’ and ‘6347’, the susceptible checks were completely dead at 14-DPI and 10-DPI, respectively. Thus, the isolate ‘PWB-186’ was the least virulent among the three; isolates ‘PWB-185’ and ‘6347’ were highly virulent, with ‘6347’ being more virulent than ‘PWB-185’. Isolate ‘6347’ was also described as the most virulent isolate (designated as ‘PWB-175’) among 13 isolates used in a previous study [49].

Breeding pepper for resistance against *P. capsici* is a challenging task due to the constant emergence of new races that can overcome the existing host resistance [50, 51]. Different resistance genes are required for different physiological races within each disease phase [10]. Race-specific resistance was observed for some of the accessions in this study. For example, ‘Floral Gem’ was resistant to isolates ‘PWB-185’ and ‘PWB-186’ but was susceptible to isolate ‘6347’. In a previous study, this cultivar was screened against 10 isolates of *P. capsici* and was found to be resistant to eight isolates and susceptible to isolates ‘PWB-24’ and ‘PWB-73’ [52]. ‘Paladin’ was susceptible to all three isolates in this study, but it was resistant to three (‘PWB-53’, ‘PWB-75’, and ‘PWB-54’) out of eight isolates in a previous study [52]. ‘Paladin’ was also found to be resistant to isolates from New York and North Carolina, USA [53, 54]. In this study, ‘NuMex Vaquero’ was susceptible to all three isolates, but a previous study reported that this variety had resistance against Phytophthora root rot races 2 and 3 [55]. Five accessions, namely ‘Chilhuacle Orange’, ‘Tipo Ancho’, ‘NMCA10237’, ‘13C905-6’, and ‘Tipo Pasilla’ exhibited broad-spectrum resistance and were found to be completely resistant to the three isolates used in this study. These accessions can serve as potential resistant sources for future genomic breeding aimed at developing chile pepper with Phytophthora root rot resistance.

### Population structure of the *Capsicum* spp. accessions

Genetic subpopulations for the genotypes were derived using Bayesian iterative algorithm approach used in STRUCTURE software. Based on the ad hoc Evanno criterion, the optimal number of clusters, *K*, was determined to be 7 (Fig. 3D), indicating that the population could be divided into seven subpopulations. This finding was also supported by the results of the phylogenetic analysis (Fig. 3C). Values for alpha, *α* (relative admixture levels between populations) for this *K* were also consistent among replications, and hence convergence of the implemented algorithm was verified (data not shown). About 97% of the panel used in this study consisted of *C. annuum* accessions and only four accessions were interspecific hybrids between *C. annuum* and *C. frutescens*. These four interspecific accessions formed a separate cluster based on the results from STRUCTURE, PCA, and Neighbour-joining analysis (Group E; Figs. 3B and 3C). The rest of the six clusters consisted of the *C. annuum* accessions. In previous studies, complex genetic relatedness has been found within the *C. annuum* group [32, 56], where accessions clustered into different groups. Taranto et al. [57] found clustering in *C. annuum* based on geographical locations and fruit-related traits. Similar to previous studies [58, 59], clusters of *C. annuum* accessions were detected on the basis of fruit or pod type in this study. The PCA revealed five groups, whereas the STRUCTURE analysis resulted in *K* = 7 optimum clusters; this disparity between PCA and STRUCTURE results has been previously noted in other studies [32, 58]. The optimum *K* derived in performing STRUCTURE analysis may not necessarily represent the ideal number of clusters since it is determined based on a pre-determined sampling method. Therefore, when interpreting the results of STRUCTURE, it is crucial to consider the biological significance of the optimum number of clusters [60].

The knowledge of physical distance of LD decay across the population of interest facilitates the selection of the number of markers for association studies. If LD decays over long distances, fewer markers are necessary for association mapping, resulting in lower resolution. Conversely, if LD decays rapidly within a short distance, a larger number of markers is needed for association mapping and the resolution is higher [61]. Rapid LD decay (~ 0.10 Mb) was observed for the population in this study. The marker density of one single nucleotide polymorphism (SNP) per 48.04 kb, on average, was thus sufficient for detecting the genomic loci associated with *P. capsici* root rot resistance. Our results were consistent with previous studies which detected rapid LD decay for *C. annuum* at 0.07 Mb [58] and 0.01 Mb [57]. While rapid LD decay in chile pepper would require an increased number of markers, it would also provide advantages to breeders in performing fine mapping of the genes of interest identified from association studies.

### Significant markers associated with *P. capsici* root rot resistance 

Performing multi-locus GWAS resulted in the detection of a total of 330 SNP markers with the highest being detected using the isolate ‘6347’ (114 SNPs). Fifty-six markers which were common in two or more isolates distributed across all 12 chromosomes were detected. Previous studies have also mapped the resistance loci on chromosomes 1–12 of chile pepper [8, 9, 27, 28, 62–70]. Chromosome 5 has been previously identified as a prominent region for the occurrence of resistance genes against *P. capsici* in *Capsicum* spp. [8, 9, 28, 71]. In this study, seven significant SNPs were identified on chromosome 5 with *S5_14665044* explaining the maximum phenotypic variation of up to 29.6%. This marker was located ~ 0.2 Mb upstream of a major QTL peak detected using bulk segregant analysis that covered 18.8 cM and was delimited by two markers, *CONTIG6473* and *CONTIG1896* [72]. *Phyto5SAR* marker showing highest LOD at the major QTL detected by Liu et al. [72], was located ~ 1.4 Mb downstream to the *S5_25470050* SNP identified in this study. GBS-derived-SNPs on chromosome 5 detected by Siddique et al. [9] at 27.0 to 29.5 Mb, were also located ~ 1.4 Mb downstream to the *S5_25470050.* Three SNP markers (*S5_9345710*, *S5_14665044*, and *S5_25470050*) detected using GWAS in this study coincide with the extended *Pc5.1* (*Ext-Pc5.1*) region on chromosome 5 that is located between 8.35 Mb and 38.13 Mb [73]. These SNPs also coincide with the *MQTL5.1* identified from meta-QTL analysis in chile pepper [71]. SNP *S5_207353938* detected in this study was located ~ 0.19 Mb upstream to significant SNP *S05_207549766* detected for isolate ‘KPC-7’ in another study with phenotypic variation ranging from 3.5% to 29.5% [9]. The findings of this study along with previous research provide further evidence supporting the significance of chromosome 5 for Phytophthora root rot resistance.

The SNP markers *S6_170978694* and *S12_225476747* identified in this study were ~ 0.53 Mb upstream and ~ 1.28 Mb downstream to significant SNP markers, *S06_171517874* and *S12_224191357* detected on chromosomes 6 and 12, respectively [9]. Another SNP marker, *S10_197712360* identified in this study was ~ 1.69 Mb downstream to the SNP, *S10_196014162* on chromosome 10 detected in a previous GWAS [9]. The significant marker, *S8_133105638* identified in the current GWAS on chromosome 8 was ~ 0.45 Mb downstream to *QTL.Pc8.1* detected using a RIL population obtained by crossing ‘CM-334’ (resistant accession) and ‘Early Jalapeno’ (susceptible accession) [8]. The previously identified *QTL.Pc9* was also ~ 1.91 Mb upstream to the significant SNP, *S9_237909149* detected on chromosome 9 in the present work [8]. In another GWAS, significant SNPs were identified at 9.14 Mb and 208.4 Mb on chromosomes 7 and 12 respectively, which were ~ 0.11 Mb and ~ 0.91 Mb upstream to the SNPs, *S7_9249493* and *S12_209274913* detected in this study [30]. Among the significant SNPs discussed above, only *S10_197712360* and *S12_209274913* markers were potentially associated with resistance genes based on their quantitative trait nucleotide (QTN) effect. The rest of the markers have potential association with the susceptibility genes for *P. capsici* root rot. A novel major SNP marker, *S6_705421* was discovered in this study which accounted for up to 54.4% of the phenotypic variation. Most of the significant SNPs identified in this research represent potential novel resistance loci which can be targeted in breeding toward resistance. Though chromosome 5 has been known to be the major region with disease resistance loci against *P. capsici*, this study as well as previous studies have detected genomic regions on other chromosomes which demonstrates the complexity of Phytophthora root rot resistance in chile pepper [8, 9, 27, 30, 68, 74]. SNP validation will be performed using Kompetitive allele-specific markers (KASP®). KASP represents a homogeneous genotyping technology that relies on allele-specific oligo extension and fluorescence resonance energy transfer (FRET) to generate signals. This method is recognized for its cost-effectiveness and adaptability, making it a widely employed assay on a global scale [75, 76].

### Candidate genes for Phytophthora root rot resistance

This study identified candidate genes linked to disease resistance. Nucleotide-binding site leucine-rich repeat (NBS-LRR) class resistance genes are widely recognized for conferring disease resistance. The LRR regions of these genes interact with extracellular ligands while cytoplasmic kinase domains facilitate signal transduction through phosphorylation [77]. These are essential for the proper function of pathogen recognition as they are responsible for identifying effectors that pathogens deliver to host cells during infection which leads to the activation of a strong resistance response known as effector-triggered immunity [78, 79]. A total of nine genes that encode LRR receptor-like serine/threonine-protein kinase protein was detected near SNP *S2_137881264* on chromosome 2, two genes near *S12_5016287,* and one gene near *S3_38944749*, *S4_212420204*, *S10_122768586*, and *S10_189438330*. Most resistance (*R*) proteins that play a role in detecting pathogens and triggering innate immune responses possess a central nucleotide-binding domain (NBS found in NBS-LRR proteins) [80]. The NBS domain, also known as the NB-ARC domain [81] is comprised of three subdomains, NB, ARC1, and ARC2. The NB-ARC domain performs as a functional ATPase domain, and the activity of the *R* protein is regulated by its nucleotide-binding state [80]. A total of six genes that code for the NB-ARC domain were detected near SNP *S6_705421*, *S6_6636269*, *S10_201485448*, and *S11_44892*.

Receptor-like proteins and receptor-like kinases are extracellular surface receptors that play role as pattern recognition receptors in plants. They detect the molecular patterns derived from both microbes and the host, initiating the first stage of inducible defense for plant immunity, growth, and development [9, 82]. The genes encoding these proteins were detected in this study near SNPs *S5_207353938*, *S8_141949349*, *S10_201485448*, and *S12_37348368*. These candidate genes were also previously detected and are good candidates for Phytophthora root rot resistance in chile pepper [9]. *SAR8.2* gene, also designated as *CASAR82A*, is a systemic acquired resistance (SAR)-related gene which plays role in pathogen infection, environmental stresses, and abiotic elicitors [83]. This gene was also identified previously in other *P. capsici* resistance study [9] and was also detected near SNP, *S5_14665044* in this study, suggesting its involvement in Phytophthora root rot resistance in chile pepper.

Ethylene plays a vital role in numerous developmental processes and is known to be a critical mediator of abiotic and biotic stress responses in plants [84]. Previous studies have documented the involvement of ethylene responsive transcription factors in pathogen attack [85–88]. These factors were recently identified to be upregulated in plants infected with *P. capsici* [89]. In this study, the ethylene responsive transcription factor genes were identified near *S4_206485683*, *S4_212420204*, and *S12_5016287* and might be important candidate genes involved in *P. capsici* resistance. Glucan endo-1,3-beta-glucosidases, also known as *β*-1,3-glucanases, are essential hydrolytic enzymes in pathogenesis-related groups of proteins and abundant in various plant species following pathogen infection [90]. These enzymes contribute significantly to the defense response by degrading the *β*-1,3-glucans that are present in the cell wall of microbes, especially fungus and generating signaling glucans which trigger the activation of global responses [91]. The gene coding for this enzyme detected in the proximity of SNPs *S7_9249493*, *S12_1728396*, *S12_1902119*, *S12_5016287*, and *S12_209274913* might have a crucial role in degrading the *β*-1,3-glucans that are present in the cell wall of *P. capsici* and can be a strong candidate gene for resistance in chile pepper. A few late blight resistance genes were found to be associated with SNPs *S2_137881264* (1), *S6_705421* (4), *S6_6636269* (1), *S11_44892* (3), and *S12_225476747* (1). These genes also belong to NB-ARC-domains containing resistance genes and encode a specific domain of potato resistance genes [92]. The plant cell wall serves as the initial physical barrier against pathogen intrusion and plays role in detecting external signals and stimulating defense response [93, 94]. The candidate genes associated with cell wall biosynthesis, organization, and modification like hydroxyproline-rich glycoproteins (*S2_137881264*, *S8_140731657*), UDP-glucuronic acid decarboxylase (*S3_29930687*, *S5_9345710*), and *β*-D-xylosidase (*S1_65866380*, *S4_212420204*), were identified which can play role in defense response against *P. capsici*. Previous reports have also identified the involvement of epigenetic mechanisms in the defense response to *P. capsici* infection [8, 71, 73]. In this study, candidate genes for DNA methylation, histone methylation, acetylation, and ubiquitination were detected, supporting the argument for the involvement of the epigenome in defense response. Overall, many candidate genes involved in different mechanisms were detected reflecting the complex genetic architecture of resistance to *P. capsici* root rot in chile pepper. Further molecular analysis needs to be conducted to better understand and confirm their roles in conferring root rot disease resistance in *Capsicum*.

## Conclusions

Multi-locus GWAS was used to detect the genomic regions linked to Phytophthora root rot resistance. A total of 330 significant markers were identified using various multi-locus GWAS models on five disease parameters and three *P. capsici* isolates (‘PWB-185’, ‘PWB-186’, and ‘6347’). Overall, 56 significant markers distributed across all 12 chromosomes were found to be common across the isolates. Candidate genes including nucleotide-binding site leucine-rich repeat (NBS-LRR), receptor-like kinase (RLKs), system acquired resistance (*SAR8.2*), *β*-1,3-glucanases, late blight resistance genes, and genes involved in epigenetic mechanisms like histone methylation were identified. Results from this study will be further used for validation using Kompetitive allele-specific markers (KASP®) and can be used for marker-assisted selection and genomic selection for *P. capsici* resistance in *Capsicum*.

## Materials and methods

### Plant material

In this study, a diverse panel of *Capsicum* spp., comprising 157 accessions, including 44 cultivars, 99 breeding lines, and 14 landraces, was used (Table S5). The plants were grown in trays with 15 accessions and three checks in each tray at the Fabián García Research Center Greenhouse, New Mexico State University (NMSU), Las Cruces, NM (32°16′46.7″N, 106°46′24.7″W), under standard conditions for growing chile pepper [95]. Three replications were planted to screen for each isolate, where a replication consisted of up to four plants per accession. The checks used for the isolate ‘PWB-185’ were ‘CM-334’ (landrace; resistant check), ‘Camelot’ (bell pepper; susceptible check 1), and ‘NMCA 10399’ (jalapeño; susceptible check 2). For isolates ‘PWB-186’ and ‘6347’, all checks were the same as for ‘PWB-185’ isolate but ‘Ninja’ (bell pepper) was instead used in place of ‘Camelot’ as susceptible check 1 due to the lack of seeds.

### Inoculum preparation and infection

Zoospore production was performed based on previous methods [8, 46, 96]. The isolates were grown on V8^®^ media petri plate. The V8 media (500 mL) consisted of calcium carbonate (1.5 g), technical agar (10 g), V8 juice (81.5 ml), distilled water (418 ml), rifampicin (0.2 g), ampicillin (0.25 g) and primarcin (100 µL). These plates were used to grow the *P. capsici* isolates for 5–7 days at room temperature. Then, under a fume hood, 5 cm plugs were excised from these petri dishes using a cork borer. A total of 5–7 of these plugs were placed in deep petri dishes (~ 200) with 25 mL of sterile distilled water. The formation of sporangia was induced by transferring the deep dishes to an incubator at 25 °C for 48 h with fluorescent lighting. Zoospore release was induced by transferring these dishes to a 4 °C cold chamber for 60 min and then placing them back in the incubator for 30 min. A cheesecloth was used to filter the contents of the deep petri dishes to remove agar plugs. The resulting solution was subjected to zoospore quantification using a hemocytometer (Hausser Scientific, PA, USA). The final concentration of zoospores was adjusted using sterile distilled water to ~ 2,000 zoospores per ml.

**Fig. 5:**
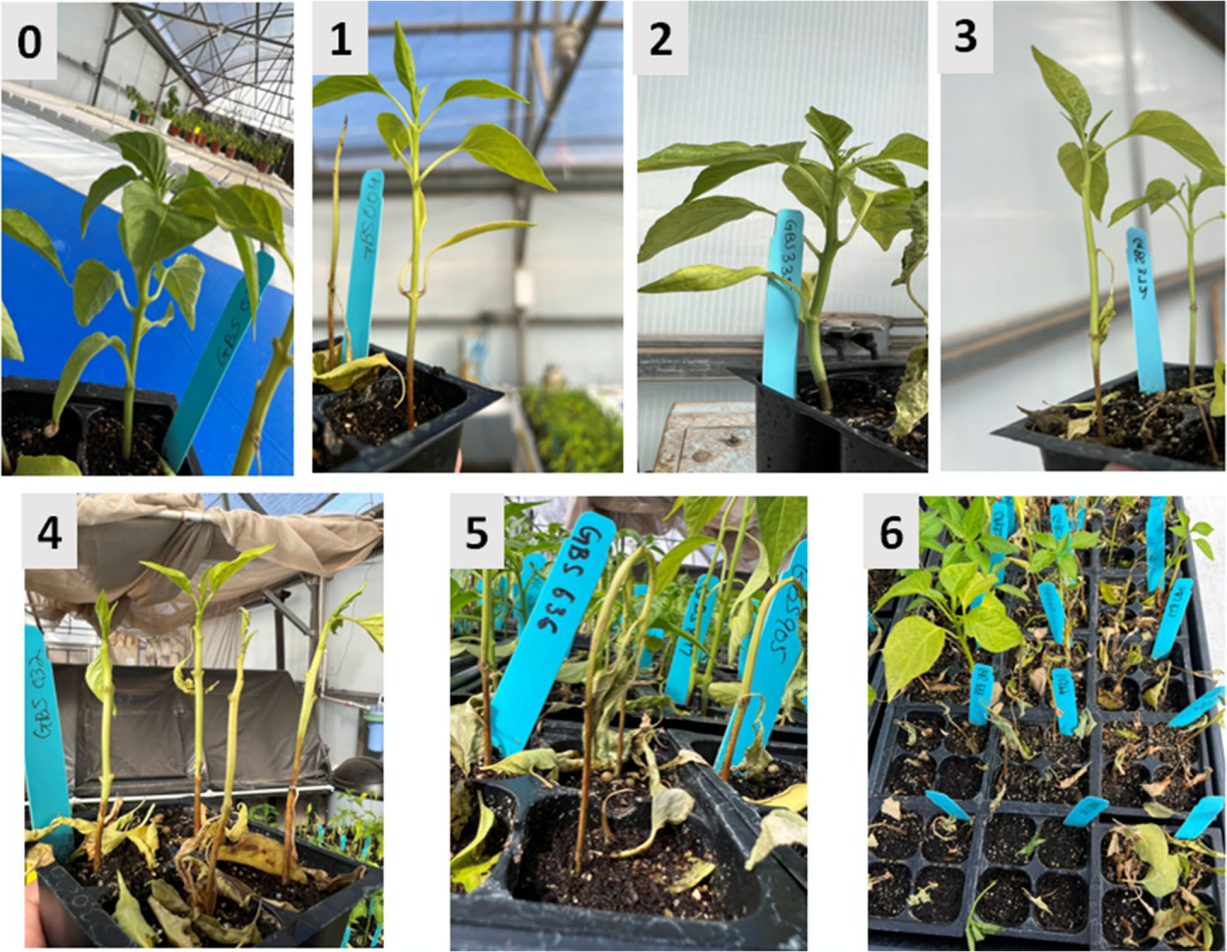
Disease score ratings (0–6) for screening chile pepper plants for resistance to *Phytophthora capsici* root rot. Ratings: 0 = no visible disease symptoms, 1 = stem necrosis with no girdling, 2 = stem necrosis with girdling, 3 = stem necrosis with less than 50% defoliation, 4 = stem necrosis with greater than 50% defoliation, 5 = wilted, and 6 = dead [8, 46]

At the 4–8 leaf stage, inoculation was done by dispensing 5 mL of the final solution of 2,000 zoospores per mL to the soil in each plant cell resulting in ~ 10,000 zoospores for each plant. Disease scoring for the inoculated plants was done after every two or three days starting at three days post-inoculation (DPI) up to 14-DPI. A disease scoring system of 0–6 was used, where 0 = no visible disease symptoms, 1 = stem necrosis with no girdling, 2 = stem necrosis with girdling, 3 = stem necrosis with less than 50% defoliation, 4 = stem necrosis with greater than 50% defoliation, 5 = wilted, and 6 = dead (Fig. 5) [8, 46].

### Phenotypic data collection and analysis

Initially, a total of 174, 166, and 164 accessions were screened for ‘PWB-185’, ‘PWB-186’, and ‘6347’ respectively, but only 157 accessions that were common among the three isolates were used for further analysis. The accessions were screened using a randomized complete block design (RCBD) with three replications for each isolate. Each block contained 15 accessions and three checks (one resistant and two susceptible checks), and each accession had a total of four plants. The four disease scores obtained for each replication of each accession were averaged to obtain a single disease score value. Screening for the three isolates was done separately and disease scores for each isolate were used to calculate the area under disease progress curve, disease severity index percentage (DSI%), estimated marginal means (least square means), and percent resistant plants. The ‘audpc’ function in ‘agricolae’ [97] package in R was used to calculate the area under disease progress curve (AUDPC) using the formula AUDPC=$\sum_{i=1}^{n-1}\frac{{y}_{i}+{y}_{i+1}}{2} {\text{x}}\left({t}_{i+1}- {t}_{i}\right),$ where, *y*_*i*_ = disease score of the plants at *i*^th^ observation (*i* = 1 being the first observation at time zero), *t*_*i*_ = number of days post infection at *i*^*th*^ observation, and *n* = number of observations.

The estimated marginal (least square) means were also calculated in R using the ‘emmeans’ function in the ‘emmeans’ package and disease score at 14-DPI [98]. Disease severity index percentage and percentage of resistant plants were calculated using the SAS® OnDemand for Academics (SAS Institute, Inc., Cary, NC). The formula from Chiang et al. [99] was used to calculate the disease severity index percentage for each line: DSI% = $$\frac{[sum\left(class frequency\times score of rating class\right)]}{[\left(total number of plants\right)\times \left(maximal disease index\right)]}\times 100.$$ According to the rating scale used in this study, the maximal disease index was a score of 6 for all genotypes. Percent resistance was calculated based on using the formula from Muhyi and Bosland [100]: $$\frac{Number of plants with disease score 1 or lower}{Total number of plants}\times 100$$.

The ‘aov’ function in R was used to perform analysis of variance (ANOVA) considering DPI, block, entry, and replication as fixed effects. Dunnett tests were also performed to compare the accessions against the resistant and susceptible checks for each isolate using R [101]. The disease score distribution of each isolate for normality was tested using Shapiro–Wilk Test. Broad-sense heritability, *H*^*2*^, was calculated for resistance to each isolate of *P. capsici* using the formula: *H*^*2*^ = *σ*^*2*^_*G*_/*σ*^*2*^_*P*_, where *σ*^*2*^_*G*_ is the variance associated with the genotype and *σ*^*2*^_*P*_ is the variance associated with the phenotype.

### Genotypic data

DNA extraction was performed using single leaf tissue from each chile pepper accession aged 30–45-days. The extraction was conducted using Qiagen DNEasy® plant extraction kits at the University of Minnesota Genomics Center (UMGC) DNA extraction facility (https://genomics.umn.edu/service/dna-extraction). DNA quantification was done using Picogreen (ThermoFisher Scientific, MA, USA). The samples were normalized to a concentration of 10 ng/µl for genotyping-by-sequencing (GBS). The UMGC performed GBS utilizing a single-enzyme digestion protocol as previously described [8, 58]. In this protocol, 10 units of restriction enzyme *ApeKI* (New England Biolabs®, Inc. MA, USA) were used to digest 100 ng of DNA per sample for 2 h at 75 °C, followed by 20 min of heat inactivation at 80 °C. The DNA samples were then heat killed after being ligated for 1 h at 22 °C with 200 units of T4 ligase (New England Biolabs®, Inc. MA, USA) and phased adaptors. Purification of the ligated samples was done followed by the amplification for the addition of the barcodes. Purification, quantification, and pooling of the GBS libraries was subsequently performed. DNA fragments within the size range of 300–744 bp size region were then specifically selected and diluted to a concentration of 1 nM. These selected fragments were subjected to sequencing with the Illumina NovaSeq™ 6000 (Illumina, CA, USA) using single end 1 × 100 reads.

Illumina ‘bcl2fastq’ software was used for the demultiplexing of the FASTQ files and first 12 bases from the beginning of each read were removed using Trimmomatic to get rid of the adapter sequences [102]. The alignment of FASTQ files to the ‘Zunla-1’ reference genome [103] was done using BWA (Burrows-Wheeler Aligner) [104]. To remove variants with genotype rates < 95%, minor allele frequency < 1%, and samples with genotype rates < 50%, raw VCF files were processed using VCFtools [105]. TASSEL [106, 107] was used to convert VCF files to HapMap format. Further, filtering of the HapMap file based on taxa was done and SNP markers with MAF < 0.05 and minor states were removed. Missing data imputation was done using the LD *k*-nearest neighbor genotype imputation (LD-*k*nni) function [108] in TASSEL. ‘CMplot’ package in R was used to construct the SNP density plot per chromosome [109].

### Population structure and linkage disequilibrium analysis

A total of 55,117 markers were used to identify the subpopulations within the diversity panel using an admixture model in STRUCTURE 2.3.4 [60]. The length of burn-in period and number of Monte Carlo Markov Chain (MCMC) replicates after burn-in were selected as 10,000 and the number of replications per *K* was set to 5. The number of clusters, *K*, were defined between 1 and 10 and the optimal *K* for the population was inferred using the STRUCTURE HARVESTER program (https://github.com/dentearl/structureHarvester/) [110, 111]. Convergence of the algorithm was verified by comparing the average values for alpha, *α* (i.e., relative admixture levels between populations for each replication per *K*). TASSEL 5.2.86 software was used to perform principal component analysis (PCA) to analyze the population structure where five major components were considered for further analysis. PCA biplot using the first two PCs and scree plot were constructed using R. TASSEL was also used to construct a neighbor-joining (NJ) phylogenetic tree which was further edited in iTOL (Interactive Tree of Life) website (https://itol.embl.de/) [112]. The PCA biplot and NJ tree were based on the optimum clusters obtained from STRUCTURE results. The “Sliding window” type function in TASSEL with a window size of 50 (0.05 Kb) was used to perform linkage disequilibrium (LD) analysis [106]. The “ggplot2” package in R was used to plot LD decay over distance using the TASSEL results [113].

### Genomewide association study

A multi-locus genomewide mapping approach was used to detect significant loci linked with area under disease progress curve (AUDPC), disease severity index percentage (DSI%), percent resistant plants, and estimated marginal means for the three *P. capsici* isolates. The ‘lme4’ package in R was used to calculate the Best Linear Unbiased Estimates (BLUEs) [114]. Best Linear Unbiased Predictors (BLUPs) were computed considering the three isolates as different “environments” and performing combined analyses using the same package in R. Overall, three different BLUE phenotypic values (‘PWB-185’, ‘PWB-186’, ‘6347’), and a BLUP (‘PWB-185_PWB-186_6347’) for the traits were used for GWAS.

The multi-locus random-SNP effect mixed linear model (‘mrMLM.GUI’) package in R was used to perform GWAS [115]. Six multi-locus GWAS models, namely, Multi-locus random-SNP-effect mixed linear model (mrMLM) [116], Iterative modified-sure independence screening Expectation–Maximization-Bayesian least absolute shrinkage and selection operator (ISIS EM-BLASSO) [117], Polygenic-background-control based least angle regression plus empirical Bayes (pLARmEB) [118], Fast multi-locus random-SNP-effect efficient mixed model association (FASTmrEMMA) [119], Fast multi-locus random-SNP-effect mixed linear model (FASTmrMLM) [120], and Polygenic Kruskal–Wallis method with Empirical Bayes (pKWmEB) [121] were used with the default parameters. The GWAS model included five principal components and a kinship matrix (*K-PC*) to address population structure. A threshold of 3.0 LOD score was employed to declare markers as statistically significant in the GWAS models.

### Candidate gene analysis

Candidate gene analysis was performed using EnsemblPlants [122], whereby genes located within 0.5 Mb of the SNP marker were considered as the candidate genes. An annotation file for ‘Criollo de Morellos-334’ (‘CM-334’; Genome assembly (GA)/ ASM512225v2) (*C. annuum* L.) was downloaded from the EnsemblPlants website (https://ftp.ensemblgenomes.ebi.ac.uk/pub/plants/release-55/gff3/capsicum_annuum/) [122, 123]. The list of candidate genes and their annotations for each significant SNP marker was subsequently derived from the annotation file using R. Candidate gene analyses were performed for significant markers that were common across all phenotypic datasets.

### Supplementary Information

Supplementary Material 1.

Supplementary Material 2.

Supplementary Material 3.

Supplementary Material 4.

Supplementary Material 5.

## Data Availability

Disease scores and marker information used in this study is available from the Authors upon reasonable request.

## Abbreviations

ANOVA: Analysis of variance
AUDPC: Area under disease progress curve
BLUE: Best linear unbiased estimates
BLUP: Best linear unbiased predictors
DPI: Days post inoculation
DSI%: Disease severity index percentage
GBS: Genotyping-by-sequencing
GWAS: Genome-wide association study
KASP: Kompetitive allele specific PCR
LD: Linkage disequilibrium
MCMC: Markov chain Monte Carlo
NBS-LRR: Nucleotide-binding site leucine-rich repeat
PCA: Principal component analysis
QTN: Quantitative trait nucleotide
RCBD: Randomized complete block design
SAR8.2: Systemic acquired resistance 8.2
RLK: Receptor-like kinase
SNP: Single nucleotide polymorphisms
UMGC: University of Minnesota Genomics Center

## References

[1] Leonian LH (1922). Stem and fruit blight of peppers caused by *Phytophthora capsici* sp. Nov. Phytopathology.

[2] Barchenger DW, Lamour KH, Bosland PW (2018). Challenges and strategies for breeding resistance in *Capsicum annuum* to the multifarious pathogen *Phytophthora capsici*. Front Plant Sci.

[3] Sanogo S, Lamour K, Kousik S, Lozada DN, Parada Rojas CH, Quesada-Ocampo L, et al. *Phytophthora capsici*, 100 Years Later: Research mile markers from 1922 to 2022. Phytopathology. 2022. 10.1094/PHYTO-08-22-0297-RVW. 10.1094/PHYTO-08-22-0297-RVW 36401843

[4] Cheng W, Lin M, Chu M, Xiang G, Guo J, Jiang Y, et al. RNAi-based gene silencing of RXLR effectors protects plants against the oomycete pathogen *Phytophthora capsici*. Molecular Plant-Microbe Interactions. 2022;35:440–9. 10.1094/MPMI-12-21-0295-R. 10.1094/MPMI-12-21-0295-R 35196108

[5] Erwin DC, Ribeiro OK. Phytophthora: diseases worldwide. Minnesota, US: APS Press. 1996. 10.1046/j.1365-3059.1998.0179a.x.

[6] Walker SJ, Bosland PW (1999). Inheritance of Phytophthora root rot and foliar blight resistance in pepper. J Am Soc Hortic Sci.

[7] Lamour KH, Stam R, Jupe J, Huitema E (2012). The oomycete broad-host-range pathogen *Phytophthora capsici*. Mol Plant Pathol.

[8] Lozada DN, Nunez G, Lujan P, Dura S, Coon D, Barchenger DW (2021). Genomic regions and candidate genes linked with
* Phytophthora capsici
* root rot resistance in chile pepper (
* Capsicum annuum
* L.). BMC Plant Biol.

[9] Siddique MI, Lee H-Y, Ro N-Y, Han K, Venkatesh J, Solomon AM (2019). Identifying candidate genes for *Phytophthora capsici* resistance in pepper (*Capsicum annuum*) via genotyping-by-sequencing-based QTL mapping and genome-wide association study. Sci Rep.

[10] Monroy-Barbosa A, Bosland PW (2008). Genetic analysis of Phytophthora root rot race-specific resistance in chile pepper. J Am Soc Hortic Sci.

[11] Sy O, Bosland PW, Steiner R (2005). Inheritance of phytophthora stem blight resistance as compared to phytophthora root rot and phytophthora foliar blight resistance in *Capsicum annuum* L. J Am Soc Hortic Sci.

[12] Sy O, Steiner R, Bosland P (2008). Recombinant inbred line differential identifies race-specific resistance to Phytophthora root rot in *Capsicum annuum*. Phytopathology.

[13] Reeves G, Monroy-Barbosa A, Bosland PW (2013). A novel *Capsicum* gene inhibits host-specific disease resistance to *Phytophthora capsici*. Phytopathology.

[14] Monroy-Barbosa A, Bosland PW (2010). A rapid technique for multiple-race disease screening of phytophthora foliar blight on single
* Capsicum annuum *L. plants. HortScience.

[15] Ben-Chaim A, Borovsky Y, Falise M, Mazourek M, Kang B-C, Paran I (2006). QTL analysis for capsaicinoid content in *Capsicum*. Theor Appl Genet.

[16] Han K, Lee H, Ro N, Hur O, Lee J, Kwon J (2018). QTL mapping and GWAS reveal candidate genes controlling capsaicinoid content in *Capsicum*. Plant biotechnol J.

[17] Lee J, Park SJ, Hong SC, Han J, Choi D, Yoon JB (2016). QTL mapping for capsaicin and dihydrocapsaicin content in a population of *Capsicum annuum* ‘NB 1’× *Capsicum chinense* ‘Bhut Jolokia’. Plant Breeding.

[18] Dwivedi N, Kumar R, Paliwal R, Kumar U, Kumar S, Singh M (2015). QTL mapping for important horticultural traits in pepper (*Capsicum annuum* L.). J Plant Biochem Biotechnol.

[19] Rao G, Ben Chaim A, Borovsky Y, Paran I (2003). Mapping of yield-related QTLs in pepper in an interspecific cross of *Capsicum annuum* and *C. frutescens*. Theor Appl Genet.

[20] Chaim AB, Paran I, Grube R, Jahn M, Van Wijk R, Peleman J (2001). QTL mapping of fruit-related traits in pepper (*Capsicum annuum*). Theor App Genet..

[21] Han K, Jeong H-J, Yang H-B, Kang S-M, Kwon J-K, Kim S (2016). An ultra-high-density bin map facilitates high-throughput QTL mapping of horticultural traits in pepper (*Capsicum annuum*). DNA Res.

[22] Wei J, Li J, Yu J, Cheng Y, Ruan M, Ye Q (2020). Construction of high-density bin map and QTL mapping of horticultural traits from an interspecific cross between *Capsicum annuum* and Chinese wild *Capsicum frutescens*. Biotechnol Biotechnol Equip.

[23] Yarnes SC, Ashrafi H, Reyes-Chin-Wo S, Hill TA, Stoffel KM, Van Deynze A (2013). Identification of QTLs for capsaicinoids, fruit quality, and plant architecture-related traits in an interspecific *Capsicum* RIL population. Genome.

[24] Li N, Yin Y, Wang F, Yao M (2018). Construction of a high-density genetic map and identification of QTLs for cucumber mosaic virus resistance in pepper (*Capsicum annuum* L.) using specific length amplified fragment sequencing (SLAF-seq). Breed Sci.

[25] Yao M, Li N, Wang F, Ye Z (2013). Genetic analysis and identification of QTLs for resistance to cucumber mosaic virus in chili pepper (*Capsicum annuum* L.). Euphytica.

[26] Mallard S, Cantet M, Massire A, Bachellez A, Ewert S, Lefebvre V (2013). A key QTL cluster is conserved among accessions and exhibits broad-spectrum resistance to *Phytophthora capsici*: a valuable locus for pepper breeding. Mol Breed.

[27] Naegele R, Ashrafi H, Hill T, Chin-Wo SR, Van Deynze A, Hausbeck M (2014). QTL mapping of fruit rot resistance to the plant pathogen *Phytophthora capsici* in a recombinant inbred line *Capsicum annuum* population. Phytopathology.

[28] Rehrig WZ, Ashrafi H, Hill T, Prince J, Van Deynze A (2014). CaDMR1 cosegregates with QTL Pc5. 1 for resistance to *Phytophthora capsici* in pepper (*Capsicum annuum*). Plant Genome..

[29] Zhu C, Gore M, Buckler ES, Yu J. Status and prospects of association mapping in plants. Plant Genome. 2008;1. 10.3835/plantgenome2008.02.0089.

[30] Ro N, Haile M, Hur O, Geum B, Rhee J, Hwang A, et al. Genomewide association study of resistance to *Phytophthora capsici* in the pepper (*Capsicum* spp.) collection. Front Plant Sci. 2022:1615. 10.3389/fpls.2022.902464. 10.3389/fpls.2022.902464 PMC9164128 35668797

[31] Zhang Y-M, Jia Z, Dunwell JM. Editorial: The applications of new multi-locus GWAS methodologies in the genetic dissection of complex traits. Front Plant Sci. 2019;10. 10.3389/fpls.2019.00100. 10.3389/fpls.2019.00100 PMC6378272 30804969

[32] Lozada DN, Barchenger DW, Coon D, Bhatta M, Bosland PW (2022). Multi-locus association mapping uncovers the genetic basis of yield and agronomic traits in chile pepper (*Capsicum* spp.). CBGG.

[33] Malik P, Kumar J, Singh S, Sharma S, Meher PK, Sharma MK (2021). Single-trait, multi-locus and multi-trait GWAS using four different models for yield traits in bread wheat. Mol Breed.

[34] Peng Y, Liu H, Chen J, Shi T, Zhang C, Sun D (2018). Genome-wide association studies of free amino acid levels by six multi-locus models in bread wheat. Front Plant Sci.

[35] Vikas V, Pradhan AK, Budhlakoti N, Mishra DC, Chandra T, Bhardwaj S (2022). Multi-locus genome-wide association studies (ML-GWAS) reveal novel genomic regions associated with seedling and adult plant stage leaf rust resistance in bread wheat (*Triticum aestivum* L.). Heredity.

[36] Ma L, Liu M, Yan Y, Qing C, Zhang X, Zhang Y (2018). Genetic dissection of maize embryonic callus regenerative capacity using multi-locus genome-wide association studies. Front Plant Sci.

[37] Xu Y, Yang T, Zhou Y, Yin S, Li P, Liu J (2018). Genome-wide association mapping of starch pasting properties in maize using single-locus and multi-locus models. Front Plant Sci.

[38] Zhang Y, Liu P, Zhang X, Zheng Q, Chen M, Ge F (2018). Multi-locus genome-wide association study reveals the genetic architecture of stalk lodging resistance-related traits in maize. Front Plant Sci.

[39] Ikram M, Han X, Zuo J-F, Song J, Han C-Y, Zhang Y-W (2020). Identification of QTNs and their candidate genes for 100-seed weight in soybean (*Glycine max* L.) using multi-locus genome-wide association studies. Genes.

[40] Li S, Xu H, Yang J, Zhao T (2019). Dissecting the genetic architecture of seed protein and oil content in soybean from the Yangtze and Huaihe River valleys using multi-locus genome-wide association studies. Int J Mol Sci.

[41] Zhang K, Liu S, Li W, Liu S, Li X, Fang Y (2018). Identification of QTNs controlling seed protein content in soybean using multi-locus genome-wide association studies. Front Plant Sci.

[42] Misra G, Badoni S, Domingo CJ, Cuevas RPO, Llorente C, Mbanjo EGN (2018). Deciphering the genetic architecture of cooked rice texture. Front Plant Sci.

[43] Zhong H, Liu S, Sun T, Kong W, Deng X, Peng Z (2021). Multi-locus genome-wide association studies for five yield-related traits in rice. BMC Plant Biol.

[44] Dang VH, Hill CB, Zhang X-Q, Angessa TT, McFawn L-A, Li C (2022). Multi-locus genome-wide association studies reveal novel alleles for flowering time under vernalisation and extended photoperiod in a barley MAGIC population. Theor Appl Genet.

[45] Hu X, Zuo J, Wang J, Liu L, Sun G, Li C (2018). Multi-locus genome-wide association studies for 14 main agronomic traits in barley. Front Plant Sci.

[46] Sanogo S (2004). Response of chile pepper to *Phytophthora capsici *in relation to soil salinity. Plant Dis.

[47] Brachi B, Morris GP, Borevitz JO (2011). Genome-wide association studies in plants/ the missing heritability is in the field. Genome biol.

[48] Stakman EC, Stewart D, Loegering WQ (1962). Identification of physiologic races of Puccinia graminis var. tritici.

[49] Jiang L, Sanogo S, Bosland PW (2015). Using recombinant inbred lines to monitor changes in the race structure of *Phytophthora capsici* in chile pepper in New Mexico. Plant Health Prog..

[50] Barchenger DW, Sheu Z-M, Kumar S, Lin S-W, Burlakoti RR, Bosland PW (2018). Race characterization of Phytophthora root rot on *Capsicum* in Taiwan as a basis for anticipatory resistance breeding. Phytopathology.

[51] Boutemy LS, King SR, Win J, Hughes RK, Clarke TA, Blumenschein TM (2011). Structures of Phytophthora RXLR effector proteins: a conserved but adaptable fold underpins functional diversity. J Biol Chem.

[52] Oelke LM, Bosland PW, Steiner R (2003). Differentiation of race specific resistance to Phytophthora root rot and foliar blight in *Capsicum annuum*. J Am Soc Hortic Sci.

[53] Dunn AR, Lange HW, Smart CD (2014). Evaluation of commercial bell pepper cultivars for resistance to phytophthora blight (*Phytophthora capsici*). Plant Health Prog..

[54] Parada-Rojas CH, Quesada-Ocampo LM (2019). Characterizing sources of resistance to phytophthora blight of pepper caused by *Phytophthora capsici* in North Carolina. Plant Health Prog..

[55] Bosland PW. ‘NuMex Vaquero’Jalapeno. HortScience. 2010;45:1552–3. 10.21273/HORTSCI.45.10.1552.

[56] Parry C, Wang Y-W, Lin S, Barchenger DW (2021). Reproductive compatibility in *Capsicum* is not necessarily reflected in genetic or phenotypic similarity between species complexes. PLoS ONE.

[57] Taranto F, D’Agostino N, Greco B, Cardi T, Tripodi P (2016). Genome-wide SNP discovery and population structure analysis in pepper (*Capsicum annuum*) using genotyping by sequencing. BMC Genomics.

[58] Lozada DN, Bhatta M, Coon D, Bosland PW (2021). Single nucleotide polymorphisms reveal genetic diversity in New Mexican chile peppers (*Capsicum* spp). BMC Genomics.

[59] Gonzalez-Perez S, Garces-Claver A, Mallor C, de SaenzMiera LE, Fayos O, Pomar F (2014). New insights into *Capsicum* spp relatedness and the diversification process of *Capsicum annuum* in Spain. PLoS ONE.

[60] Pritchard JK, Stephens M, Donnelly P (2000). Inference of population structure using multilocus genotype data. Genetics.

[61] Abdurakhmonov IY, Abdukarimov A. Application of association mapping to understanding the genetic diversity of plant germplasm resources. Int J Plant Genomics. 2008;2008. 10.1155/2008/574927. 10.1155/2008/574927 PMC2423417 18551188

[62] Kim H-J, Nahm S-H, Lee H-R, Yoon G-B, Kim K-T, Kang B-C (2008). BAC-derived markers converted from RFLP linked to *Phytophthora capsici* resistance in pepper (*Capsicum annuum* L.). Theor Appl Genet.

[63] Lu F-H, Kwon S-W, Yoon M-Y, Kim K-T, Cho M-C, Yoon M-K (2012). SNP marker integration and QTL analysis of 12 agronomic and morphological traits in F8 RILs of pepper (*Capsicum annuum* L.). Mol Cells.

[64] Minamiyama Y, Tsuro M, Kubo T, Hirai M (2007). QTL analysis for resistance to *Phytophthora capsici* in pepper using a high density SSR-based map. Breed Sci.

[65] Ogundiwin EA, Berke TF, Massoudi M, Black LL, Huestis G, Choi D (2005). Construction of 2 intraspecific linkage maps and identification of resistance QTLs for *Phytophthora capsici* root-rot and foliar-blight diseases of pepper (*Capsicum annuum* L.). Genome.

[66] Quirin E, Ogundiwin E, Prince J, Mazourek M, Briggs M, Chlanda T (2005). Development of sequence characterized amplified region (SCAR) primers for the detection of Phyto. 5.2, a major QTL for resistance to *Phytophthora capsici* Leon. in pepper. Theor Appl Genet.

[67] Sugita T, Yamaguchi K, Kinoshita T, Yuji K, Sugimura Y, Nagata R (2006). QTL analysis for resistance to Phytophthora blight (*Phytophthora capsici* Leon.) using an intraspecific doubled-haploid population of *Capsicum annuum*. Breed Sci.

[68] Thabuis A, Palloix A, Pflieger S, Daubeze A-M, Caranta C, Lefebvre V (2003). Comparative mapping of Phytophthora resistance loci in pepper germplasm: evidence for conserved resistance loci across Solanaceae and for a large genetic diversity. Theor Appl Genet.

[69] Truong H, Kim K, Kim D, Kim S, Chae Y, Park J (2012). Identification of isolate specific resistance to Phytophthora root rot in pepper (*Capsicum annuum* L.). Plant Pathol.

[70] Xu X, Chao J, Cheng X, Wang R, Sun B, Wang H (2016). Mapping of a novel race specific resistance gene to Phytophthora root rot of pepper (*Capsicum annuum*) using bulked segregant analysis combined with specific length amplified fragment sequencing strategy. PLoS ONE.

[71] Lozada DN, Whelpley M, Acuña-Galindo A (2021). Genetic architecture of chile pepper (*Capsicum* spp.) QTLome revealed using meta-QTL analysis. Hortic.

[72] Liu W-Y, Kang J-H, Jeong H-S, Choi H-J, Yang H-B, Kim K-T (2014). Combined use of bulked segregant analysis and microarrays reveals SNP markers pinpointing a major QTL for resistance to *Phytophthora capsici* in pepper. Theor Appl Genet.

[73] Du J-S, Hang L-F, Hao Q, Yang H-T, Ali S, Badawy RSE (2021). The dissection of R genes and locus Pc5 1 in *Phytophthora capsici* infection provides a novel view of disease resistance in peppers. BMC Genomics.

[74] Bonnet J, Danan S, Boudet C, Barchi L, Sage-Palloix A-M, Caromel B (2007). Are the polygenic architectures of resistance to *Phytophthora capsici* and *P. parasitica* independent in pepper?. Theor Appl Genet.

[75] Kumpatla SP, Buyyarapu R, Abdurakhmonov IY, Mammadov JA. Genomics-assisted plant breeding in the 21st century: technological advances and progress. INTECH Open Access Publisher, Den Haag. 2012. http://www.intechopen.com/books/plantbreeding/genomics-assisted-plant-breeding-in-the-21stcenturytechnological-advances-and-progress.

[76] Semagn K, Babu R, Hearne S, Olsen M (2014). Single nucleotide polymorphism genotyping using Kompetitive Allele Specific PCR (KASP): overview of the technology and its application in crop improvement. Mol Breeding.

[77] Andersen EJ, Ali S, Byamukama E, Yen Y, Nepal MP (2018). Disease resistance mechanisms in plants. Genes.

[78] Heidrich K, Blanvillain-Baufumé S, Parker JE (2012). Molecular and spatial constraints on NB-LRR receptor signaling. Curr Opin Plant Biol.

[79] Lee S, Choi D (2013). Comparative transcriptome analysis of pepper (*Capsicum annuum*) revealed common regulons in multiple stress conditions and hormone treatments. Plant Cell Rep.

[80] Van Ooijen G, Mayr G, Kasiem MM, Albrecht M, Cornelissen BJ, Takken FL (2008). Structure–function analysis of the NB-ARC domain of plant disease resistance proteins. J Exp Bot.

[81] McHale L, Tan X, Koehl P, Michelmore RW (2006). Plant NBS-LRR proteins: adaptable guards. Genome Biol.

[82] Tang D, Wang G, Zhou J-M (2017). Receptor kinases in plant-pathogen interactions: more than pattern recognition. Plant Cell.

[83] Lee S, Hwang B (2003). Identification of the pepper SAR8. 2 gene as a molecular marker for pathogen infection, abiotic elicitors, and environmental stresses in Capsicum annuum. Planta.

[84] Müller M, Munné-Bosch S (2015). Ethylene response factors: a key regulatory hub in hormone and stress signaling. Plant Physiol.

[85] Cillo F, Palukaitis P. Transgenic resistance. In: Advances in virus research. Elsevier; 2014. p. 35–146. 10.1016/B978-0-12-801246-8.00002-0. 10.1016/B978-0-12-801246-8.00002-0 25410101

[86] Davies HA, Daniels MJ, Dow JM (1997). Induction of extracellular matrix glycoproteins in Brassica petioles by wounding and in response to *Xanthomonas campestris*. Mol Plant-Microbe Interac.

[87] Peumans WJ, Van Damme E (1995). Lectins as plant defense proteins. Plant Physiol.

[88] Holland KW, FO’keefe S (2010). Recent applications of peanut phytoalexins. Recent Pat Food Nutr Agric.

[89] Bagheri LM, Nasr-Esfahani M, Abdossi V, Naderi D (2020). Analysis of candidate genes expression associated with defense responses to root and collar rot disease caused by *Phytophthora capsici* in peppers *Capsicum annuum*. Genomics.

[90] Kebede A, Kebede M (2021). In silico analysis of promoter region and regulatory elements of glucan endo-1, 3-beta-glucosidase encoding genes in *Solanum tuberosum*: cultivar DM 1–3 516 R44. J Genet Eng Biotechnol.

[91] Perrot T, Pauly M, Ramírez V (2022). Emerging roles of β-glucanases in plant development and adaptative responses. Plants.

[92] Chunthawodtiporn J, Hill T, Stoffel K, Van Deynze A. Genetic analysis of resistance to multiple isolates of *Phytophthora capsici* and linkage to horticultural traits in bell pepper. HortScience. 2019;54:1143–8. 10.21273/HORTSCI13359-18.

[93] Cosgrove DJ (2005). Growth of the plant cell wall. Nat Rev Mol Cell Biology..

[94] Wan J, He M, Hou Q, Zou L, Yang Y, Wei Y (2021). Cell wall associated immunity in plants. Stress Biol..

[95] Sharma H, Shukla MK, Bosland PW, Steiner R (2017). Soil moisture sensor calibration, actual evapotranspiration, and crop coefficients for drip irrigated greenhouse chile peppers. Agric water manag.

[96] Bosland PW, Lindsey D (1991). A seedling screen for Phytophthora root rot of pepper *Capsicum annuum*. Plant Dis.

[97] De Mendiburu F (2014). ‘Agricolae’: statistical procedures for agricultural research. R package version.

[98] Lenth R, Lenth MR (2018). Package ‘lsmeans’. Am Stat.

[99] Chiang K, Liu H, Bock C (2017). A discussion on disease severity index values. Part I: warning on inherent errors and suggestions to maximise accuracy. Ann Appl Biol.

[100] Muhyi R, Bosland PW. Evaluation of *Capsicum* germplasm for sources of resistance to *Rhizoctonia solani*. HortScience. 1995;30:341–2. 10.21273/HORTSCI.30.2.341.

[101] R Core Team. R: A language and environment for statistical computing. R Foundation for Statistical Computing, Vienna, Austria. 2013. http://www.r-project.org/.

[102] Bolger AM, Lohse M, Usadel B (2014). Trimmomatic: a flexible trimmer for Illumina sequence data. Bioinform.

[103] Qin C, Yu C, Shen Y, Fang X, Chen L, Min J (2014). Whole-genome sequencing of cultivated and wild peppers provides insights into *Capsicum* domestication and specialization. Proc Nat Acad Sci..

[104] Li H, Durbin R (2009). Fast and accurate short read alignment with Burrows-Wheeler transform. Bioinform.

[105] Danecek P, Auton A, Abecasis G, Albers C, Banks E, Durbin R (2011). The variant call format and VCFtools. Bioinform.

[106] Bradbury PJ, Zhang Z, Kroon DE, Casstevens TM, Ramdoss Y, Buckler ES (2007). TASSEL: software for association mapping of complex traits in diverse samples. Bioinform.

[107] Glaubitz JC, Casstevens TM, Lu F, Harriman J, Elshire RJ, Sun Q (2014). TASSEL-GBS: a high-capacity genotyping by sequencing analysis pipeline. PLoS ONE.

[108] Money D, Gardner K, Migicovsky Z, Schwaninger H, Zhong G-Y, Myles S. LinkImpute: fast and accurate genotype imputation for nonmodel organisms. G3: Genes, Genomes, Genet. 2015;5:2383–90. 10.1534/g3.115.021667. 10.1534/g3.115.021667 PMC4632058 26377960

[109] Package YL, “CMplot”.  (2019). R Foundation for Statistical Computing.

[110] Earl DA, VonHoldt BM (2012). Structure Harvester: a website and program for visualizing Structure output and implementing the Evanno method. Conserv Genet Res.

[111] Evanno G, Regnaut S, Goudet J (2005). Detecting the number of clusters of individuals using the software STRUCTURE: a simulation study. Mol Ecol.

[112] Letunic I, Bork P (2021). Interactive Tree Of Life (iTOL) v5: an online tool for phylogenetic tree display and annotation. Nucleic Acids Res.

[113] Wickham H, Chang W, Wickham MH (2016). Package ‘ggplot2’. Create elegant data visualisations using the grammar of graphics Version.

[114] Bates D, Mächler M, Bolker B, Walker S. Fitting linear mixed-effects models using ‘lme4’. arXiv preprint arXiv:14065823. 2014. 10.48550/arXiv.1406.5823.

[115] Ya-Wen Z, Pei L, Yuan-Ming Z, Zhang MY, Package ‘mrMLM. GUI’.  (2019). R Foundation for Statistical Computing.

[116] Wang S-B, Feng J-Y, Ren W-L, Huang B, Zhou L, Wen Y-J (2016). Improving power and accuracy of genome-wide association studies via a multi-locus mixed linear model methodology. Sci Rep.

[117] Tamba CL, Ni Y-L, Zhang Y-M (2017). Iterative sure independence screening EM-Bayesian LASSO algorithm for multi-locus genome-wide association studies. PLoS Comput Biol.

[118] Zhang J, Feng J-Y, Ni Y, Wen Y, Niu Y, Tamba C (2017). pLARmEB: integration of least angle regression with empirical Bayes for multilocus genome-wide association studies. Heredity.

[119] Wen Y-J, Zhang H, Ni Y-L, Huang B, Zhang J, Feng J-Y (2018). Methodological implementation of mixed linear models in multi-locus genome-wide association studies. Brief Bioinform.

[120] Tamba CL, Zhang Y-M. A fast mrMLM algorithm for multi-locus genome-wide association studies. biorxiv. 2018:341784. 10.1101/341784.

[121] Ren W-L, Wen Y-J, Dunwell JM, Zhang Y-M (2018). pKWmEB: integration of Kruskal-Wallis test with empirical Bayes under polygenic background control for multi-locus genome-wide association study. Heredity.

[122] Bolser D, Staines DM, Pritchard E, Kersey P. EnsemblPlants: integrating tools for visualizing, mining, and analyzing plant genomics data. In: Plant bioinformatics. Springer; 2016. p. 115–40. 10.1007/978-1-4939-3167-5_6. 10.1007/978-1-4939-3167-5_6 26519403

[123] Kim S, Park M, Yeom S-I, Kim Y-M, Lee JM, Lee H-A (2014). Genome sequence of the hot pepper provides insights into the evolution of pungency in *Capsicum* species. Nat genet.

